# Psychosocial symptoms of ventricular arrhythmias: Integrating patient-reported outcomes into clinical care

**DOI:** 10.1016/j.hroo.2021.09.011

**Published:** 2021-12-17

**Authors:** Uday Sandhu, Adrienne H. Kovacs, Babak Nazer

**Affiliations:** Knight Cardiovascular Institute, Oregon Health & Science University, Portland, Oregon

**Keywords:** Implantable cardioverter-defibrillator, Patient-reported outcomes, Patient-reported outcome measures, Psychological counseling, Sudden cardiac death/arrest, Ventricular arrhythmias

## Abstract

Patient-reported outcome measures (PROMs) are a valuable metric for assessing the quality of life and overall well-being in patients with ventricular arrhythmias (VAs) and/or implantable cardioverter-defibrillators (ICDs). The incorporation of PROMs into the workflow of a VA clinic not only allows for more patient-centered care but also may improve detection and treatment of clinically relevant anxiety or depression symptoms. Awareness of the factors known to correlate with adverse PROM scores may guide PROM administration and subsequent referral to mental health services. Further, change or stability in PROM scores can be used as a gauge to guide the effectiveness of cardiac and psychological treatment in certain populations that are the focus of this manuscript: patients with ICDs (with and without shocks), cardiac arrest survivors, and those with inherited arrhythmia syndromes.

## Graphical abstract

**Figure:**
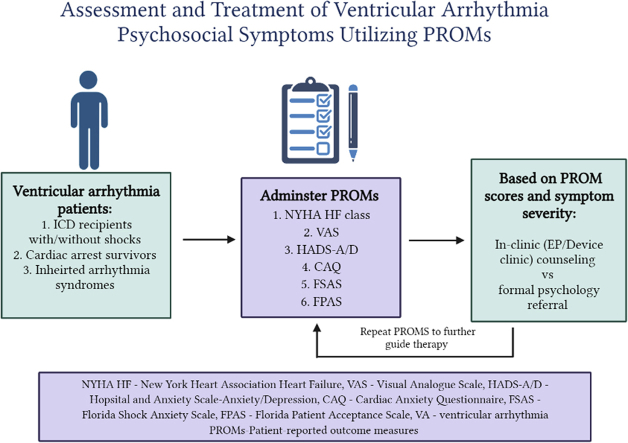

**Key Findings**

▪ Patient-reported measure outcomes (PROMs) are validated questionnaires that are valuable in evaluating patients’ quality of life, functional status, and mental health.
▪ Patients with ventricular arrhythmias are at risk for psychosocial symptoms of anxiety and depression, leading to poor quality of life.
▪ Utilizing PROMs to guide psychological treatment for patients with ventricular arrhythmias can improve their functional outcomes. Additional randomized data are required to confirm clear improvement in PROMs with psychological interventions.

## Introduction

Pharmacologic, ablation, and implantable device–based treatments for ventricular arrhythmias (VAs) have grown considerably, with a priority on reducing morbidity and mortality. However, there is a growing need for providers to address the quality of life (QOL) and psychosocial well-being of patients with VAs, particularly those who have experienced or are at risk for cardiac arrest or implantable cardioverter-defibrillator (ICD) shocks. This requires assessment *from the perspective of patients*, not providers. Patient-reported outcomes (PROs) are defined as “any report of the status of a patient’s health condition that comes directly from the patient, without interpretation of the patient’s response by a clinician or anyone else.”^1^ Patient-reported outcome measures (PROMs) are validated questionnaires that quantify aspects of the patient experience, including symptoms, functional status, mental health, and QOL.

PROMs were originally primarily used within a research context and are gradually being incorporated within health systems in order to assess symptom severity, inform treatment decisions, facilitate patient-provider discussions, monitor health and well-being, and connect providers to patient-generated health data.^2^ PROMs can also be employed in comparing effectiveness of therapies to determine which treatments result in the greatest benefit for patients, and as a metric for value-based payment.^3^ As such, the Centers for Medicare and Medicaid Services, National Quality Forum, and European Society of Cardiology have all formally called to promote PROM development, validation, and use in cardiovascular care and research.^3^^,^^4^

The overarching objective of this manuscript is to provide guidance to electrophysiology (EP) programs wishing to integrate PRO assessments, particularly for 4 categories of VA patients: cardiac arrest survivors, ICD recipients (with a focus on patients who receive shocks), those who undergo ventricular tachycardia (VT) ablation, and patients with inherited arrhythmia syndromes.

## Cardiac arrest survivors

Survival to hospital discharge for out-of-hospital cardiac arrest (OHCA), a significant portion of which are due to VAs, increased from 10.2% in 2006 to 12.4% in 2015.^5^ Unsurprisingly, surviving an OHCA often carries significant and lasting physical and psychosocial impact. A recent integrative review of survivors of cardiopulmonary rehabilitation concluded that although QOL may often be acceptable, symptoms of depression, anxiety, post-traumatic stress disorder (PTSD), “avoidance behavior,” cognitive dysfunction, fatigue, and an inability to return to work are common.^6^ Published rates of clinically significant anxiety range from 13% to 61%^6^, ^7^, ^8^ and depression from 14% to 45%.^9^, ^10^, ^11^ In a recent US cohort study of 184,568 adults with prior cardiac arrest, 24% were diagnosed with a psychiatric disorder, most commonly a mood disorder (16.4%).^12^ Wide ranges in prevalence might reflect variability in specific PROMs used to measure psychological distress as well as the duration of time since OHCA. However, a study with serial follow-up of 168 OHCA patients over the course of a year demonstrated that although physical and social function partially improved by 6 months, there were no reductions in the prevalence of depression or anxiety between 1 and 12 months postarrest.^7^ Independent predictors of post-OHCA mood disorders include female sex, younger age, White ethnicity, poorer general health, and pre-OHCA trauma exposure.^12^^,^^13^

There is emerging evidence that psychoeducational interventions can improve outcomes following OHCA. A randomized controlled trial (RCT) demonstrated that a psychosocial intervention achieved a reduction in risk of cardiovascular death of 86% (*P* = .03).^14^ This intervention consisted of 11 individual sessions that included physiologic relaxation with biofeedback training, cognitive-behavioral therapy, and cardiovascular health education. Although there was no improvement in anxiety, a significant effect of the intervention on depression was noted. It should be noted this was small trial with a sample size of 129 with 7 cardiovascular deaths in the control group and 1 in the treatment group. Another RCT of a brief nursing intervention targeting the emotional and cognitive sequelae of sudden cardiac arrest revealed positive outcomes regarding QOL and mental health at 1-year follow-up, with 39% more patients returning to work.^15^

In cardiology and EP clinics, an awareness of the high prevalence of post-OHCA psychological distress supports a systematic approach to (1) either routine or selective (based on the risk factors above) use of PROMs to identify individuals experiencing clinically significant depression, anxiety, and/or PTSD, and (2) providing mental health treatment by a practitioner who understands the unique and enduring challenges associated with surviving a significant acute medical event that approximately 90% of the population does not survive.

## ICD recipients

Prevalence of anxiety and depression in ICD recipients has been reported by a meta-analysis to be 11%–28% with diagnostic interviews and 5%–63% based on PROMs, representing the large variability among studies.^16^ The effects of ICD implant on QOL, anxiety, and depression in RCTs of ICD implantation such as CABG-Patch, CIDS, AVID, and SCD-HeFT have been mixed.^17^^,^^18^ Meta-analyses have demonstrated that ICD implant is not associated with lower QOL scores when compared to medical therapy.^17^^,^^19^^,^^20^ Furthermore, there appear to be trends toward improved patient satisfaction and QOL with remote monitoring compared to in-clinic follow-up.^21^

However, despite ICD recipients as a group not clearly having a reduction in QOL, anxiety and depression can develop in a subset of patients, even in the absence of shocks. Risk factors that have been associated with anxiety and/or depression postimplant include younger age,^22^ female sex,^22^^,^^23^ lack of knowledge of ICD and illness,^24^ unmarried or unpartnered with low social support,^22^^,^^25^ type D personalities (defined as a tendency toward negative affect and social inhibition),^26^ and additional medical comorbidities.^27^, ^28^, ^29^

“Device acceptance” has been defined as “the psychological accommodation and understanding of the device and the derivation of benefit in terms of biopsychosocial functioning.”^25^ As assessed by the Florida Patient Acceptance Scale (FPAS) PROM, rates of acceptance are lower in the setting of pre-existing anxiety and depression, symptomatic heart failure, and type D personality.^25^ Having a partner/spouse correlated with greater device acceptance.^30^

Randomized trials of psychosocial interventions for ICD-associated anxiety and depression have included in-person cognitive behavioral stress management therapy,^31^ group and telephone counseling,^32^ and ICD patient education programs,^33^ all of which demonstrated modest reductions in anxiety and/or depression symptoms as measured by the PROMs. A meta-analysis of RCTs evaluated the effect of psychoeducational interventions on physical and mental components of patients’ health status, and found that only the physical component (aggregated from short form [SF]-36/SF-12 scores) of the patient’s QOL improved, with no significant difference in the mental component.^34^

Prior to ICD implant, cardiologists should be mindful of risk factors for psychological distress leading to poor device acceptance, including those listed above. Patients who report or exhibit significant ICD-related anxiety should be offered for preimplantation psychological therapy (in advance of elective procedures). Also, the physician should make apparent to the patient, especially in the setting of primary prevention ICDs, that an ICD will reduce the risk of sudden cardiac death from VAs but will not treat the underlying etiology of sudden cardiac death, and an implant may lead to new potential sources of anxiety.^35^ Following ICD implant, psychosocial well-being and device acceptance can be serially assessed using the FPAS PROM, identifying patients who may benefit from ongoing ICD education and support from their physician and device clinic team, with referral to mental health professionals as needed.

## Individuals who receive ICD shocks

More than half of patients receiving ICDs for secondary prevention experience ICD shocks in long-term follow-up.^36^ The incidence is less common among primary prevention ICD recipients, particularly in the era of high rate and/or long delay programming, although the MADIT-RIT study still demonstrated annual shock incidence of 6.4% (3.5% appropriate, 2.9% inappropriate shocks).^37^ Device shocks may carry an independent burden of anxiety, depression, and PTSD, which can adversely affect QOL. In the CABG-PATCH trial,^38^ the reduced QOL (assessed via SF-36 PROM) in the ICD arm was restricted to those who experienced an ICD shock. The AVID trial also demonstrated lower SF-36 questionnaire scores in the setting of ICD shocks.^39^ However, the potential relationship between ICD shocks and impaired QOL is confounded, as deterioration of patients’ underlying structural heart disease could be a common etiology.^40^ Accordingly, in other ICD (including primary prevention) trials, effects of shocks on QOL have been mixed and rarely demonstrated dose-response relationships.^18^ In the CIDS trial, poorer QOL and psychological distress was noted only among patients who received 5 or more shocks,^41^ suggesting a threshold-based response. Furthermore, the adverse effects of ICD shocks on QOL may be restricted to the postshock period: PROs from the SCD-HeFT trial demonstrated reduced scores on SF-36 Mental Health Inventory PROM only when the shock occurred in the 30 days prior to PRO assessment at a follow-up visit.^18^ In addition, a follow-up of the PainFree SST trial noted a decrease in physical activity, measured via ICD accelerometer, of 23.7 minutes a day for the first 30 days after an ICD shock, with a greater number of shocks leading to greater reduction in activity.^42^ Interestingly, shock anxiety was higher among appropriate shocks vs inappropriate shocks.

The Florida Shock Anxiety Scale (FSAS) is a 10-item PROM that evaluates device-specific fears in regard to a device shock and assesses ability of the patient to cope with possible device therapy.^43^ A recent literature review assessing reliability and validity of FSAS demonstrated its usefulness across diverse populations, with a score of 3–5 indicating an appropriate referral for clinical psychological evaluation/support.^43^ Lower shock anxiety was reported when patients felt supported by healthcare professionals and had better understanding of device-related knowledge. Higher FSAS scores have also been observed in younger patients and women.^43^

Once a patient's QOL is affected by anxiety regarding ICD shocks, psychosocial therapies are needed. Sears and colleagues^44^ assessed an “ICD Stress and Shock Management Program” administered as either a 1-day workshop or 6 weekly sessions to patients who had received at least 1 shock in the previous year, demonstrating a reduction in depression and anxiety as quantitated by the SF-12 Mental Component PROM, and improved device acceptance as quantitated by FPAS PROM.^44^ Furthermore, cognitive-behavioral therapy sessions in ICD patients, when compared to usual care, resulted in reduction in anxiety as evaluated by a small RCT.^45^

Patients who suffer repeated ICD shocks from VT storm can often meet formal criteria for PTSD. One group administered multimodal psychotherapy (8–15 sessions over 1 year) to 18 ICD patients with diagnosed PTSD after VT storm (mean 19 shocks), and none of them met PTSD criteria at 1 year, with significant reductions in anxiety and avoidance behavior.^46^ In the absence of a control arm, though, it is unclear if these PTSD criteria would have resolved on their own over 12 months. Limited data on patients with “phantom shocks” (patients’ perception of an ICD shock in the absence of an actual shock) demonstrate an association with history of psychiatric disease and type D personality, whereas symptoms of depression and anxiety have no clear association with “phantom shocks.”^47^

Conversely, depression and anxiety have also been hypothesized to *increase* the risk for arrhythmias. In a study of 645 patients assessed for depression by the Center of Epidemiological Studies Depression Scale PROM, moderate-to-severe depression was associated with a 3-fold increased risk of ICD shocks, despite controlling for multiple confounders.^48^ A more direct link between psychological stress and ICD shocks was demonstrated in a study of 62 ICD patients, who noted anger during a “mental stress protocol” resulted in T wave alternans, which was independently predictive of ICD shocks prospectively at 1 year (33% compared with 4% without T wave alternans).^48^ A more recent study noted higher anxiety scores on certain PROMs was associated with increased risk of recurrent VAs, but only in males.^49^

Based on the above data, clinicians should consider screening for anxiety and depression with PROMs either routinely, or selectively among patients who have had VT storm or 5 or more ICD shocks. Patients with clinically elevated symptoms should have concerns addressed by their Device Clinic team, including a written “ICD Shock Plan” that explicitly states the criteria for which a patient should notify the clinic and/or seek medical attention after ICD shock(s). In our and others’ experience, explicit patient education surrounding ICD shocks may decrease anxiety by discussing the meaning of shocks and plans for prevention/avoidance of inappropriate shocks.^27^^,^^43^

## Individuals undergoing VT catheter ablation

Randomized trials of VT catheter ablation have demonstrated reductions of ICD shocks by 33%-73% and VT storm by 44%-70% over a 22-to-28-month follow-up.^50^, ^51^, ^52^ Given the observed relationship between ICD shocks and psychosocial PROs, it is hypothesized that a reduction of ICD shocks by VT ablation may improve PROs. In the randomized VTACH trial of ablation for postinfarct cardiomyopathy VT, the SF-36 was administered at 12 and 24 months of follow-up. The ablation group demonstrated a nonspecific trend toward improved health status across all subscales, although interpretation was limited because follow-up data were available in only 56% of study patients.^50^ In the THERMOCOOL postmarket, nonrandomized study, symptoms of depression and anxiety were assessed prior to and 6 months after ablation. Ablation reduced the prevalence of clinically significant anxiety from 51% to 31% (*P* = .0003) and clinically significant depression from 26% to 18% (*P* = .22).^53^

There are no known published psychosocial interventions developed as an adjunct to VT ablation. However, given the high baseline Hospital Anxiety and Depression Scale – Anxiety (HADS-A) and Hospital Anxiety and Depression Scale – Depression (HADS-D) scores among VT ablation patients and the psychosocial impact of ICD shocks, adjunctive psychosocial therapy may improve PROs. Therapy may also indirectly address adherence to antiarrhythmics, anticoagulants, heart failure medications, weight loss, and exercise, all of which may improve PROs and hard clinical outcomes of VT ablation.

## Individuals with inherited arrhythmia syndromes

Often affecting pediatric or young adult patients as well as their family members, inherited arrhythmia syndromes pose unique psychosocial challenges. Parents of patients may also be affected: among families affected by congenital long QT syndrome, 61% of parents of long QT syndrome patients report a fear of their children dying.^54^ Furthermore, elevated symptoms of anxiety and depression pose significant barriers to family communication about diagnoses and genetic testing^55^ and may prevent otherwise effective and life-saving cascade genetic testing.

Arrhythmogenic right ventricular cardiomyopathy (ARVC) often presents in young patients and is often accompanied by prescribed exercise restrictions to prevent disease progression, both of which also pose unique psychosocial issues. The correlation between prescribed exercise withdrawal with worsening anxiety and depression is well described, with increased fatigue, lower self-esteem, and irritability noted, especially in patients with longer withdrawal of exercise.^56^ Particularly in the young, previously active ARVC population, adjusting to an ICD and restricting exercise can be challenging. The study authors administered 3 PROMs to a cohort of 86 ARVC patients.^57^ Patients had significant body image concerns (as determined by an FPAS subscale) along with ICD-specific and general clinical anxiety. Predictors of anxiety were younger age, poorer functional capacity, previous ICD shocks, and shorter time since ICD implant.^57^

Catecholaminergic polymorphic ventricular tachycardia is often triggered by physical or emotional stress and, like ARVC, is often treated with exercise restriction. A small cross-sectional survey study reported the significant psychosocial effects of this disorder^58^ and found that patients under 40 years old reported higher device- and disease-related anxiety, depression, and PTSD.

Genetic arrhythmia syndromes’ unique aspects of phenotypic uncertainty, young age of affected patients, family dynamics, and prescribed exercise restriction pose psychological risks at every clinical step: prediagnosis, disease progression, treatment, and screening/surveillance of family members. The routine use of PROMs may be useful for screening and monitoring of psychological symptoms, which may otherwise go undiagnosed amidst ICD interrogation, genetic testing, and medication management. Incorporating dedicated psychological counseling into genetic arrhythmia clinics and centers will provide tailored treatment for anxiety and depression, likely improving PROs, device acceptance, medication and exercise compliance, and clinical outcomes.

## Conclusion and practical integration

Patients with or at risk of VAs may have reduced QOL and/or elevated anxiety and depression symptoms, which can be quantified using PROMs. In our practice, we administer a battery of PROMs (several of those in Table 1) at the first and every follow-up clinic visit. In addition to meeting with the EP and ICD/device team, our VA patients meet with a psychologist at their initial visit. We also incorporate PROMs into clinical decision-making, including frequency of psychology follow-up, or how quickly to wean antiarrhythmic medications after an initially successful VT ablation. Table 2 summarizes psychological interventions we routinely utilize, as well as those that warrant further research. If psychotropic medical therapy is deemed appropriate, we commonly involve the patient’s primary care physician or refer to a psychiatrist for adjunctive psychopharmacologic treatment. Review of psychopharmacology is beyond the scope of this article, but this topic has previously been reviewed in the context of cardiovascular drug-drug interactions and cardiotoxic/proarrhythmic effects.^59^

**Table 1:** Selected psychosocial patient-reported measure outcomes of utility in ventricular arrhythmia patients

Name	Number of items	Scoring	Comments
Visual Analogue Scale of physical status and quality of life	2	0–100	Patient asked to draw a line or arrow along a 0–100 scale to reflect status (higher score suggests greater quality).
Short-Form 36 (SF-36)	36	0–100	Higher score indicates better QOL.
State-Trait Anxiety Inventory (STAI)	40	20–80; none/low anxiety (20–37), moderate anxiety (38–44), high anxiety (45–80)	Higher score reflects greater anxiety.
Hospital Anxiety and Depression Scale – Anxiety (HADS-A)	7	0–21; mild (8–10), moderate (11–14), severe (15–21)	Useful for initial screening and progression. Higher score reflects greater anxiety.
Hospital Anxiety and Depression Scale (HADS-D)	7	0–21; mild (8–10), moderate (11–14), severe (15–21)	Higher score reflects increased depressive symptoms.
Center for Epidemiological Studies – Depression Scale (CES-D)	20	0–60; mild (16–20), moderate (21–25), severe (26–60)	Higher score reflects increased depressive symptoms.
Cardiac Anxiety Questionnaire (CAQ)	18	0–72	Higher score reflects greater anxiety.
Florida Patient Acceptance Scale (FPAS)	18	18–90	Higher score reflects greater ICD acceptance.
Florida Shock Anxiety Scale (FSAS)	10	10–50; none-minimal (10–20), mild (21–30), moderate (31–40), severe (41–50)	Higher score reflects greater anxiety.

ICD = implantable cardioverter-defibrillator.

**Table 2:** Summary of ventricular arrhythmia psychosocial challenges and interventions

VA disease process	Psychosocial burden	Current interventions	Future direction – research	Future direction – clinical
Cardiac arrest survivors	• Anxiety • Depression • PTSD • Avoidance behavior	• CBT • Biofeedback • Cardiovascular education	• Identify predictors of post-OHCA anxiety, depression, PTSD • Further randomized trials of psychotherapy	• Routine use of PROMs to screen and monitor psychosocial symptoms • Target psychotherapy to patients with high-risk PROM scores, and those with risk factors for post-OHCA psychosocial illness (female, younger, neurocognitive deficits, pre-OHCA trauma)
ICD recipients	• Anxiety • Depression • Poor device acceptance	• Optimize patient education • CBT	• Develop ICD-specific mental health therapies/programs • Determine if these programs reduce arrhythmia burden	• Screen for and target psychotherapy to patients at risk of poor device acceptance: younger, female, no spouse/partner, type D personality, pre-existing anxiety/depression, poor knowledge of ICD or illness, symptomatic HF
ICD shocks	• PTSD • Anxiety • Depression • Avoidance behavior • Phantom shocks	• Provide patients an explicit “ICD Shock Plan” • Psychotherapy	• Develop and study psychosocial interventions that effectively reduce symptoms and incidence of recurrent arrhythmias/shocks	• Screen ICD patients for psychosocial stress-induced ECG changes, which may be a trigger for shocks
Inherited arrhythmia syndromes	• Anxiety • Depression • ICD acceptance • Body image	• Routine clinical care	• Define psychosocial effects of exercise restriction	• Routine use of PROMs • Target psychotherapy to high-risk PROM scores, younger patients, recent ICD implants, and patients with uncertain diagnoses • Family counseling

CBT = cognitive behavioral therapy; ECG = electrocardiographic; HF = heart failure; ICD = implantable cardioverter-defibrillator; OHCA = out-of-hospital cardiac arrest; PROM = patient-reported outcome measures; PTSD = post-traumatic stress disorder; VA = ventricular arrhythmia.

**Figure 1:**
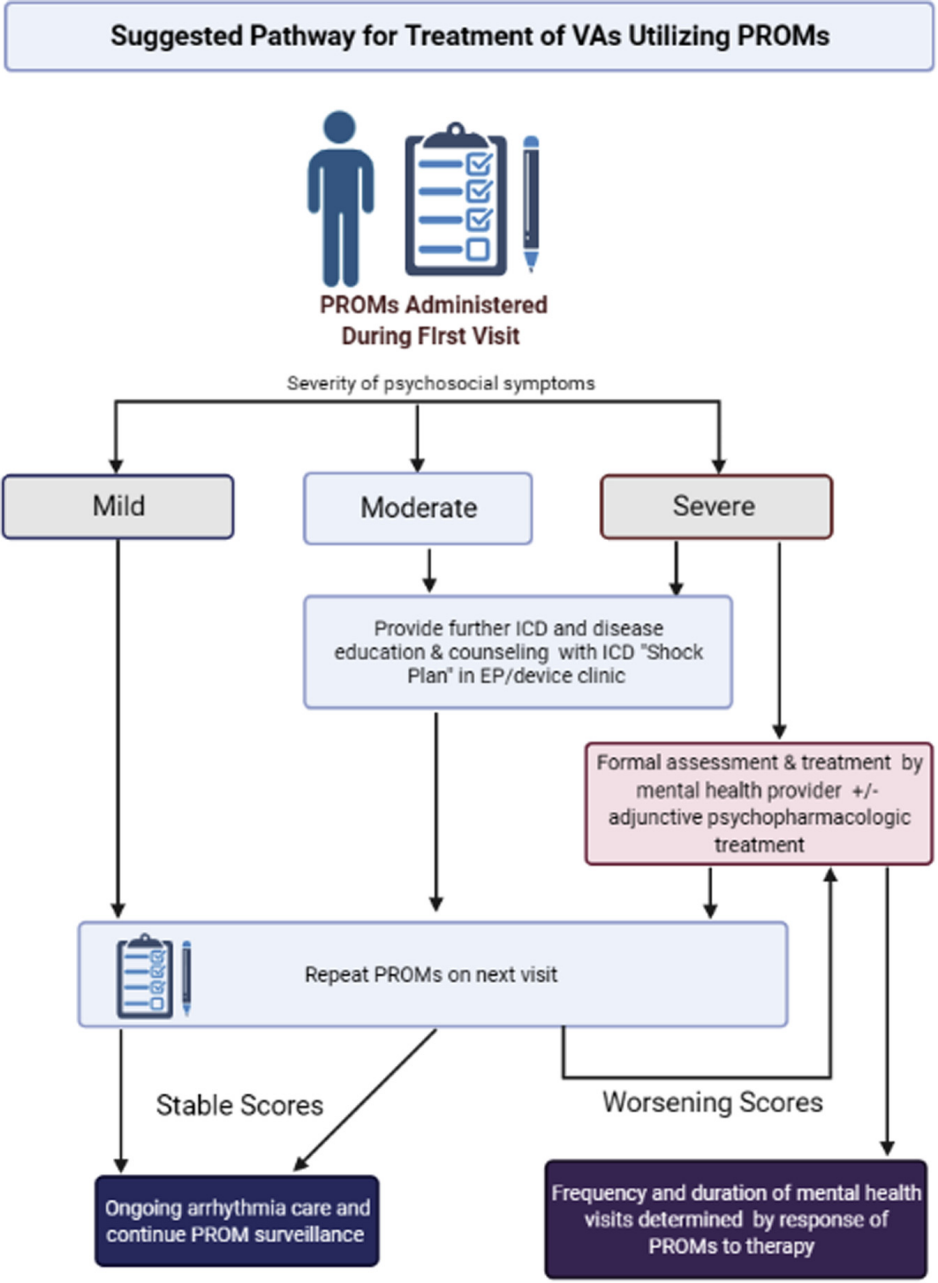
Treatment pathway for anxiety and depression symptoms detected by routine administration of patient-reported outcome measures (PROMs). EP = electrophysiology; ICD = implantable cardioverter-defibrillator; VA = ventricular arrhythmia.

Although our practice has every patient seen by a psychologist integrated into our clinic, this may not be available in all clinics, so other practices can utilize PROMs to triage patients appropriately. In these situations, routine administration of PROMs can identify patients who will benefit from more intensive support beyond simple counseling and education by their EP and Device Clinic (Figure 1), thereby triggering referral. Failure of PROM scores to improve in follow-up could also trigger referral to counseling for selected patients. A proposed treatment algorithm by Sears and colleagues is based on FSAS score: 0–20 score requires no to minimal intervention; those with scores of 21–30 are provided ICD education and a “device shock plan”; those with scores 31–40 have biweekly to monthly psychological treatment with coping skills training and teaching adaptive behaviors; and those with scores of 41–50 should have weekly psychological treatment with addition of possible adjunctive pharmacological therapies.^43^

By administering a wide battery of PROMs (Table 1), EP providers learn about both the patient’s general physical health status and QOL, while simultaneously narrowing down to specific ICD- and shock-related concerns. Specific PROM items within a certain questionnaire can also be individually analyzed, identifying specific sources of patients’ symptoms, such as a concern that increased physical activity will result in ICD shocks, as is frequently noted in our cohort of patients.^60^

Practically, PROMs can be completed by patients while in the clinic waiting room, during Device Clinic interrogations, or between sequential clinic appointments. A common barrier noted with use of paper PROM data has been the cumbersome and time-consuming manual scoring process. For these reasons, we have integrated PROMs to be completed via an electronic medical record–enabled tablet, automatically scored, and data uploaded into the electronic medical record.^61^^,^^62^

Future studies should demonstrate if formal psychological counseling yields a robust improvement in PROM scores. Studies should also determine the subset of VA patients (perhaps based on their baseline PROM scores) most likely to benefit from psychological counseling. Finally, limited data suggest that VA patients have interest in support groups,^63^ but more data on their effects on psychosocial symptoms and PROM scores is needed.
